# *EGFR*突变的晚期非小细胞肺癌的抗PD-1/PD-L1治疗研究进展

**DOI:** 10.3779/j.issn.1009-3419.2022.101.44

**Published:** 2022-10-20

**Authors:** 悦 朱, 朝霞 戴

**Affiliations:** 116021 大连，大连医科大学附属第二医院 The Second Affiliated Hospital of Dalian Medical University, Dalian 116021, China

**Keywords:** 肺肿瘤, 免疫治疗, 肿瘤微环境, Lung neoplasms, Immune therapy, Tumor microenvironment

## Abstract

表皮生长因子受体（epidermal growth factor receptor, EGFR）酪氨酸激酶抑制剂（tyrosine kinase inhibitor, TKI）是*EGFR*突变的晚期非小细胞肺癌（non-small cell lung cancer, NSCLC）的一线治疗方案，但1年-2年内会出现耐药，后续治疗效果差。程序性死亡受体1（programmed cell death 1, PD-1）/程序性死亡配体1（programmed cell death ligand 1, PD-L1）抑制剂的出现极大地改变了肿瘤治疗的格局。然而，单药抗PD-1/PD-L1对*EGFR*突变的晚期NSCLC低应答或无应答，如何使*EGFR*突变的晚期NSCLC患者从抗PD-1/PD-L1治疗中获益是需要攻克的难关。本文主要就近5年来*EGFR*突变对NSCLC免疫状态影响的研究进展及相关的临床研究进行综述。

## Abstract (en)

The use of epidermal growth factor receptor (EGFR) tyrosine kinase inhibitor (TKI) is the first line treatment for *EGFR*-mutant advanced non-small cell lung cancer (NSCLC), but drug resistance will be acquired within 1-2 years, and the following treatment efficacy is poor. The invention of programmed cell death 1 (PD-1)/programmed cell death ligand 1 (PD-L1) inhibitors has dramatically changed the situation of tumor treatment. PD-1/PD-L1 inhibitors are less effective in patients with NSCLC harboring *EGFR* mutation. It is a challenge to make patients with *EGFR*-mutated advanced NSCLC benefit from anti-PD-1/PD-L1 therapy. In this paper, the research progress on the impact of *EGFR* mutation on the immune status of NSCLC and related clinical studies in recent 5 years are reviewed.

肺癌是全球发病率第二、死亡率居首位的肿瘤^[1]^，而非小细胞肺癌（non-small cell lung cancer, NSCLC）约占85%。表皮生长因子受体（epidermal growth factor receptor, *EGFR*）突变的肺腺癌患者在欧洲人群中仅占15%，而在亚裔人群中约占50%^[2]^。程序性死亡受体1（programmed cell death 1, PD-1）/程序性死亡配体1（programmed cell death ligand 1, PD-L1）抑制剂的出现，使晚期NSCLC患者的5年生存率提高至15%以上^[3, 4]^。从近年来国内外已发表的研究来看，国内针对*EGFR*突变的NSCLC研究数量略多于国外，*EGFR*突变的NSCLC患者对抗PD-1/PD-L1治疗低应答或无应答，而且部分国内外关于抗PD-1/PD-L1的临床研究将*EGFR*突变的患者排除在外，这些现象一定程度上表明了*EGFR*突变对NSCLC患者抗PD-1/PD-L1治疗的负面影响。但也有个例报道*EGFR*突变的NSCLC从抗PD-1/PD-L1治疗获益较为显著^[5, 6]^。目前在真实世界中该类患者如何从抗PD-1/PD-L1治疗中获益，或筛选出抗PD-1/PD-L1治疗的获益人群是急需解决的问题。本文就此问题回顾了免疫治疗的机制，梳理了*EGFR*突变对NSCLC免疫环境和功能影响及相关临床研究进展，并展望未来的研究及治疗方向。

## 1 抗PD-1/PD-L1治疗机制

免疫系统主要通过效应T细胞的杀伤作用来对抗肿瘤细胞，肿瘤通过免疫逃逸来避开效应T细胞的杀伤作用^[7]^。

PD-1参与免疫反应的调节，是免疫检测点的抑制性受体，在T细胞、B细胞、髓细胞和自然杀伤（natural killer, NK）细胞上表达^[8, 9]^。PD-1的配体包括PD-L1和程序性死亡配体2（programmed cell death ligand 2, PD-L2）两种，T细胞、B细胞、巨噬细胞、内皮细胞和肿瘤细胞主要表达PD-L1，而PD-L2主要在树突状细胞（dendritic cell, DC）和巨噬细胞中表达^[10]^。T细胞受体（T cell receptor, TCR）与主要组织相容性复合体（major histocompatibility complex, MHC）的相互作用是T细胞激活的关键步骤，PD-1与其配体PD-L1、PD-L2结合后可以减弱TCR/CD28信号传导、抑制T细胞的激活，从而造成肿瘤免疫逃逸^[11, 12]^。抗PD-1/PD-L1可以竞争PD-1与PD-L1的相互作用位点，从而抑制了PD-1与PD-L1的结合，这对T细胞的活化有促进作用，从而使T细胞发挥抗肿瘤功能。

## 2 *EGFR*突变影响NSCLC免疫状态

### 2.1 肿瘤微环境（tumor microenvironment, TME）

TME的构成极为复杂，包含肿瘤细胞、免疫细胞、细胞因子、基质细胞等其他成分。一般来说，促进杀伤肿瘤的细胞有：CD4^+^ T细胞、CD8^+^ T细胞、CD134^+^细胞、CD137^+^细胞等，抑制杀伤肿瘤的细胞有调节性T细胞（regulatory T cells, Treg）、肿瘤相关巨噬细胞（tumor associated macrophages, TAMs）、髓源性抑制细胞（myeloid derived suppressor cells, MDSCs）等，各类成分相互作用对抗PD-1/PD-L1治疗产生了不同的应答。有学者^[13]^将肿瘤免疫微环境（tumor immune microenvironment, TIME）分为3个类型：①免疫炎症型：TME中同时存在CD4^+^和CD8^+^ T细胞，常伴有骨髓细胞和单核细胞，免疫细胞与肿瘤细胞易相互作用，对抗PD-1/PD-L1治疗有良好的临床效应；②免疫排除型：虽存在大量免疫细胞，但免疫细胞不能很好地浸润到肿瘤实质与肿瘤细胞相互作用，所以在抗PD-1/PD-L1治疗后，相关T细胞可活化增殖，但无浸润，临床反应差；③免疫沙漠型：环境中缺乏T细胞，对抗PD-1/PD-L1很难应答。

国内外均有研究证实对比野生型的EGFR肿瘤，*EGFR*突变的NSCLC的肿瘤微环境中有较低的免疫浸润^[14-18]^。Zhao等^[19]^通过对190例肺腺癌样本分析发现*EGFR*突变患者的CD8^+^肿瘤浸润淋巴细胞（tumor-infiltrating lymphocytes, TILs）的比例显著低于野生型患者（*P*=0.026），并通过体外实验证实*EGFR*突变的NSCLC细胞系外泌体可促进CD8^+^ T细胞凋亡，这表明通过抑制肿瘤细胞外泌体的释放可能会减少CD8^+^ TILs凋亡，使TILs更好地发挥抗肿瘤功能，但这需要试验来证实。Chen等^[20]^研究发现肿瘤细胞中免疫球蛋白转录物4（immunoglobulin-like transcript 4, ILT4）的表达与人NSCLC组织细胞中的EGFR磷酸化水平存在正相关关系，ILT4的过表达通过招募M2型TAMs和减弱T细胞反应来抑制肿瘤免疫，因此抑制ILT4表达能够增强抗肿瘤免疫功能，该研究揭示了EGFR介导的肿瘤免疫逃逸的新途径，并为*EGFR*突变的NSCLC患者的免疫治疗提供了新靶点。Cho等^[18]^通过单细胞转录组分析对比发现在*EGFR*突变的NSCLC中，NOTCH-RBPJ调节网络受损影响驻留记忆CD8^+^T细胞（T_RM_）持续状态，滤泡辅助CD4^+^T细胞(T_FH_)和B细胞之间相互作用通过CXCL13-CXCR5轴减弱，该研究表明T_FH_-B-T_RM_在三级淋巴结构形成中的协同作用受损，同时T_RM_稳态失调、T_FH_-B之间相互作用减弱，是*EGFR*突变肺癌抗PD-1反应不良的基础，而保持T_RM_稳态、恢复T_FH_-B之间相互作用可以作为未来研究的治疗策略。

从上述研究不难看出*EGFR*突变的NSCLC为免疫排除型或沙漠型，而目前对相关机制研究较少，将免疫排除型或沙漠型转变为免疫炎症型，是未来研究的重点，也是使*EGFR*突变的NSCLC从抗PD-1/PD-L1获益的手段。Tu等^[21]^研究发现在*EGFR*突变的NSCLC的异种移植小鼠模型中，抗PD-L1和抗CD73联合显著抑制了肿瘤的生长，增加了肿瘤组织中浸润性CD8^+^ T细胞的数量及γ干扰素（interferon γ, IFN-γ）和肿瘤坏死因子α（tumor necrosis factor α, TNF-α）的分泌以及与炎症和T细胞功能相关的基因表达，该研究为*EGFR*突变的NSCLC免疫表型转变为免疫炎症型提供了一种潜在的策略。

### 2.2 肿瘤突变负荷（tumor mutational burden, TMB）

TMB是利用全外显子组测序或者靶向测序数据，计算每兆碱基中发生于编码区的非同义碱基置换、插入、删除突变的数量，单位为mut/Mb。

Zhang等^[14]^对245例中国NSCLC患者研究发现*EGFR*突变的肺腺癌患者的TMB水平显著降低。Li等^[22]^通过对86例Ⅰ期浸润性肺腺癌患者进行了下一代测序（next generation sequencing, NGS）发现实性为主的腺癌（solid-predominant adenocarcinoma, SPA）患者显示出较高的*LRP1B*突变率和较高的PD-L1阳性率，而*LRP1B*突变与高TMB和更好的免疫治疗反应有关，表明SPA患者可能对免疫治疗有更好的应答。Yang等^[16]^研究发现*EGFR*突变患者的TMB水平低于野生型患者，*EGFR*-*MAPK*共突变患者的TMB和PD-L1表达水平较高并且有与野生型患者相似的免疫微环境，该研究在一定程度上解释了*EGFR*突变的NSCLC患者抗PD-1/PD-L1治疗低应答或无应答的原因，同时发现了*EGFR*突变NSCLC患者抗PD-1/PD-L1治疗的潜在获益人群，这需要临床研究进一步验证。Wang等^[23]^通过对223例肺腺癌患者的术后标本分析发现*EGFR*突变患者有低TMB水平。

通常来说，TMB越高，免疫原性越强，抗PD-1/PD-L1治疗疗效更好，上述研究一定程度上解释了*EGFR*突变的NSCLC对抗PD-1/PD-L1疗效差的原因，但TMB检测周期长，要求一定的技术水平且成本高，这限制了大规模的临床使用。

### 2.3 PD-L1

Nishimura等^[24]^研究发现*EGFR*突变的肺腺癌患者，B7/CD28的共刺激免疫检查点通路被激活，但PD-1/PD-L1的共刺激免疫检查点通路受到抑制，该研究对*EGFR*突变的NSCLC患者抗PD-1/PD-L1治疗无/低应答做出了解释，同时为抗细胞毒性T淋巴细胞相关蛋白4（cytotoxic T lymphocyte associated protein 4, CTLA-4）治疗提供了依据。Luo等^[25]^发现*EGFR*突变患者的PD-L1表达显著高于野生型患者（*P*=0.001,9）。Liu等^[26]^回顾性分析247例手术切除的原发性NSCLC患者和26例晚期NSCLC患者肿瘤PD-L1的表达发现，以1%为cut-off值，野生型EGFR患者的PD-L1表达水平显著高于*EGFR*突变患者。然而，Guo等^[27]^研究发现*EGFR*突变细胞系（HCC827、HCC2935、H1975）PD-L1表达高于EGFR野生型细胞系。

结果的不一致可能与研究对象（细胞系与组织标本）的不同有关，而且目前对PD-L1的检测无一致标准，评分方法也不同。但从研究的不一致性来看，也从一定程度上说明了*EGFR*突变对PD-L1影响的复杂性，*EGFR*突变可能在多个方面来影响PD-L1的表达，未来需要就此问题进行更深层次的探索，这会就解决*EGFR*突变的NSCLC患者的抗PD-1/PD-L1治疗低或无应答问题给出新的启示。

### 2.4 EGFR酪氨酸激酶抑制剂（tyrosine kinase inhibitor, TKI）影响免疫状态

Zhou等^[28]^通过对71例*EGFR*突变的晚期肺腺癌患者接受EGFR-TKI治疗后细胞免疫功能的影响分析发现CD3、CD4和自然杀伤（natural killer, NK）细胞数量以及CD4/CD8比率均增加。Watanabe等^[29]^研究发现EGFR-TKI处理后磷酸化形式的EGFR和ERK的下调与主要组织相容性复合体Ⅰ（major histocompatibility complex Ⅰ, MHC-Ⅰ）的上调、浸润性CD8^+^ T细胞数量的增加和PD-L1的表达增加相关，表明*EGFR*突变会从MEK-ERK途径来抑制NSCLC中MHC-Ⅰ的表达，从而导致此类肿瘤对免疫治疗的低应答或无应答，靶向MEK-ERK途径能否恢复MHC-Ⅰ的表达，从而提高免疫治疗疗效，需要试验来明确。Peng等^[30]^通过对获得性EGFR-TKI耐药前后PD-L1在*EGFR*突变的NSCLC中的状态和调控机制研究发现PD-L1、细胞间质上皮转换因子（cellular-mesenchymal epithelial transition factor, c-MET）和肝细胞生长因子（hepatocyte growth factor, HGF）之间存在正相关，这三种物质增加了淋巴细胞的PD-L1表达并减弱了淋巴细胞的活化和细胞毒性，PD-L1的下调可恢复淋巴细胞的部分细胞毒性。Ntzifa等^[31]^发现奥希替尼患者耐药时外周血的PD-L1水平升高。Kawana等^[32]^通过评估21例肺腺癌患者来研究EGFR-TKI耐药与淋巴细胞浸润之间的相关性，使用miRNA PCR阵列找出耐药前后变化最大的miRNA：miRNA-1（miR-1），发现EGFR-TKI耐药后，CD8^+^ T细胞的浸润水平显著降低，miR-1显著抑制EGFR-TKI的作用和细胞因子的诱导，从而抑制单核细胞迁移。

从以上研究不难看出EGFR-TKI的使用，会增加*EGFR*突变的NSCLC患者TME的免疫细胞浸润及PD-L1的表达，而这些研究结果给使用抗PD-1/PD-L1的时机提供了新的思路：EGFR-TKI耐药后抗PD-1/PD-L1是否会给患者带来临床获益？目前国内外少有EGFR-TKI治疗前后TMB表达情况的相关研究。

## 3 *EGFR*突变的晚期NSCLC抗PD-1/PD-L1治疗

### 3.1 抗PD-1/PD-L1的单药治疗

Mazieres等^[33]^研究发现在125例接受抗PD-1/PD-L1单药治疗的*EGFR*突变的晚期NSCLC患者中，其中位无进展生存期（progression-free survival, PFS）为2.1个月，客观缓解率（objective response rate, ORR）为12%。类似地，研究^[34-36]^证明*EGFR*突变的NSCLC患者很难从抗PD-1/PD-L1中获益，那么如何使*EGFR*突变的NSCLC患者从抗PD-1/PD-L1治疗中获益，相关机制是未来研究的方向。

### 3.2 抗PD-1/PD-L1的联合治疗

EGFR-TKI已成为*EGFR*突变的晚期NSCLC标准一线治疗方案，多数患者会在1年-2年内耐药，后续化疗临床获益有限，临床上需要新的治疗手段来解决这种困境。目前国内外对于*EGFR*突变的晚期NSCLC抗PD-1/PD-L1的联合治疗研究主要集中于一线EGFR-TKI治疗失败后。

#### 3.2.1 联合EGFR-TKI

上述章节介绍*EGFR*突变的NSCLC患者在接受EGFR-TKI治疗后会改善其TME，理论上EGFR-TKI联合抗PD-1/PD-L1治疗会有协同作用，但是数项关于EGFR-TKI联合抗PD-1/PD-L1的临床研究^[37-42]^表明此种联合用药大大增加了不良反应的发生率，因此联合用药应慎重，优化药物结构或许可以改善这种困局。但是EGFR-TKI耐药后发生继发驱动基因改变相应的靶向药物及其他新型靶向药物联合抗PD-1/PD-L1仍有很大的探索空间。

#### 3.2.2 联合化疗

化疗药物杀伤肿瘤细胞后会释放更多的抗原，这会提高肿瘤细胞的免疫原性，而且化疗能够诱导PD-L1表达产生耐药，因此，抗PD-1/PD-L1联合化疗存在协同作用。国内外均有研究验证了在*EGFR*突变的晚期NSCLC一线EGFR-TKI耐药后抗PD-1/PD-L1联合化疗的协同作用（表 1）：Arrieta等^[43]^进行的一项开放、随机的2期临床研究中，纳入了78例含铂方案化疗后耐药的晚期NSCLC患者，其中包含25例*EGFR*突变患者，该25例患者在接受铂类化疗前使用了阿法替尼或吉非替尼，其中12例患者纳入帕博利珠单抗+多西他赛组，13例患者纳入多西他赛单药组，ORR分别为58.3%和23.1%（*P*=0.14），PFS分别为6.8个月和3.5个月（*P*=0.04）。Shen等^[44]^分析*EGFR*突变的晚期NSCLC患者接受一代/二代EGFR-TKI耐药后使用免疫治疗或免疫联合化疗的疗效，该试验纳入30例患者，其中22例患者接受免疫单药治疗，8例患者接受免疫联合化疗，免疫单药组和免疫+化疗组的ORR分别为9.1%和25.0%，免疫+化疗组有更长的PFS和总生存期（overall survival, OS）趋势，没有T790M突变的患者的PFS明显长于有该突变的患者（4.23个月 *vs* 1.70个月，*P*=0.019）。Yu等^[45]^对144例*EGFR*突变的晚期NSCLC患者EGFR-TKI治疗失败后接受化疗+抗血管生成（*n*=100）与化疗+抗PD-1/PD-L1（*n*=44）疗效进行对比分析，在亚组分析中，EGFR-TKI耐药后继发性T790M突变的患者从化疗+抗PD-1/PD-L1治疗获益的可能性低于T790M阴性患者（3.42个月 *vs* 7.63个月，*P*=0.028），对于耐药后接受化疗+抗血管生成治疗的患者，继发T790M突变与未继发T790M突变患者的中位PFS无统计学差异（中位PFS分别为5.33个月、7.46个月，*P*=0.202）。Chen等^[46]^对86例*EGFR*突变的经EGFR-TKI和化疗治疗失败的晚期NSCLC患者进行了回顾性研究，患者分为帕博利珠单抗单药组（32/86）、帕博利珠单抗+化疗组（26/86）、帕博利珠单抗+安罗替尼组（28/86），中位PFS分别为1.5个月、4.3个月、3.24个月，中位OS分别为7.41个月、14.92个月、15.97个月，ORR分别为3.1%、23.1%、21.4%。Long等^[47]^对40例*EGFR*突变的使用奥希替尼耐药的晚期NSCLC患者进行了回顾性研究，该研究中20例患者接受了化疗+抗PD-1/PD-L1治疗，20例患者接受了化疗，对比分析疗效，结果表明化疗+抗PD-1/PD-L1治疗患者的ORR显著增高（45% *vs* 25%, *P*＜0.01），中位PFS显著延长（6.4个月 *vs* 2.8个月，*P*＜0.01），化疗+抗PD-1/PD-L1治疗患者的中位OS显著长于化疗患者（12.8个月 *vs* 10.5个月，*P*＜0.01）。

**表 1:** EGFR-TKI耐药后化疗联合抗PD-1/PD-L1的临床研究 Clinical trials of chemotherapy combined with anti-PD-1/PD-L1 after EGFR-TKI resistance

Author	Study design	ORR	PFS（mon）
Oscar Arrieta^[43]^	Pembrolizuymab plus Docetaxel *vs* Docetaxel	58.3% *vs* 23.1%	6.8 *vs* 3.5
Chia-Ⅰ Shen^[44]^	Immunotherapy plus Chemotherapy *vs* Immunotherapy	25.0% *vs* 9.1%	4.23 *vs* 2.93
Xin Yu^[45]^	Chemo-immunotherapy *vs* Chemo-antiangiogenesis	29.52% *vs* 13.0%	7.59 *vs* 6.90
Ya Chen^[46]^	Pembrolizuymab *vs* Pembrolizuymab plus Chemotherapy *vs* Pembrolizuymab plus Anlotinib	3.1% *vs* 23.1% *vs* 21.4%	1.5 *vs* 4.3 *vs* 3.24
Yaping Long^[47]^	Anti-PD-1/PD-L1 plus Chemotherapy *vs* Chemotherapy	45% *vs* 25%	6.4 *vs*2.8
EGFR-TKI: epidermal growth factor receptor tyrosine kinase inhibitor; PD-1: programmed cell death 1; PD-L1: programmed cell death ligand 1; ORR: objective response rate; PFS: progression-free survival.			

Aredo等^[48]^对度伐利尤单抗用于不可切除的完成根治性化疗的Ⅲ期*EGFR*突变的NSCLC患者进行了回顾性分析，该试验纳入37例患者，其中13例患者在根治性放化疗后接受度伐利尤单抗治疗，16例患者单独完成了根治性放化疗，8例患者EGFR-TKI联合根治性放化疗；根治性放化疗+度伐利尤单抗组、单独根治性放化疗组、根治性放化疗组+EGFR-TKI组的中位PFS分别为10.3个月、6.9个月、26.1个月，6例度伐利尤单抗治疗的患者在复发后接受EGFR-TKI治疗，其中1例患者在使用奥希替尼时发展为4级肺炎，该研究不仅表明免疫联合放化疗的增效作用，还提醒注意免疫治疗序贯EGFR-TKI的安全性。Wang等^[49]^通过对纳武利尤单抗联合或不联合多西他赛对晚期NSCLC患者的疗效和毒性研究得出结论：与使用单药纳武利尤单抗相比，在铂类双药化疗失败后的NSCLC患者中，与EGFR/ALK状态无关，纳武利尤单抗和多西他赛的联合治疗均能显著改善PFS和OS，然而在该研究纳入的77例患者中仅9例患者*EGFR*/*ALK*阳性。

虽然个别研究未发现获益，但这类研究样本量普遍偏小，大体上可以看出抗PD-1/PD-L1联合化疗有获益趋势，目前有相关临床研究正在进行，相信结果会令人欣慰。

#### 3.2.3 多药联合治疗

抗血管生成药物可使肿瘤组织的血管正常化，从而增加免疫细胞在肿瘤组织的浸润，改善TME，因此抗PD-1/PD-L1联合抗血管生成治疗同样有协同作用。

IMpower150研究纳入了多线耐药的晚期非鳞NSCLC患者，*EGFR*突变的亚组分析显示ABCP组（阿替利珠单抗+贝伐珠单抗+卡铂+紫杉醇）患者的中位PFS较BCP组（贝伐珠单抗+卡铂+紫杉醇）、ACP组（阿替利珠单抗+卡铂+紫杉醇）延长（10.2个月 *vs* 6.9个月 *vs* 6.9个月），ABCP组与BCP组均得到不同程度OS的改善^[50]^。Yang等^[51]^对31例*EGFR*突变的晚期NSCLC获得性EGFR-TKI耐药并接受免疫治疗的患者进行了回顾性分析，发现联合免疫治疗（免疫+化疗、免疫+抗血管生成治疗、免疫+化疗+抗血管生成治疗）比单药免疫治疗PFS略长，但无明显差异（3.42个月 *vs* 1.61个月，*P*=0.078），然而在免疫治疗前接受抗血管生成药物的患者获得更好的PFS（3.42个月 *vs* 1.58个月，*P*=0.027）。ORINET-31研究^[52]^纳入了444例EGFR-TKI耐药的晚期NSCLC患者，随机1:1:1分为信迪利单抗+IBI305（贝伐珠单抗生物类似药）+培美曲塞+顺铂组、信迪利单抗+培美曲塞+顺铂组、培美曲塞+顺铂组，从目前数据来看，对比培美曲塞+顺铂组，信迪利单抗+IBI305+培美曲塞+顺铂组的PFS显著延长（6.9个月 *vs* 4.3个月，*P*＜0.000,1）。

在*EGFR*突变的晚期NSCLC患者中，抗PD-1/PD-L1联合抗血管生成治疗对比抗PD-1/PD-L1单药使患者有不同程度的获益，然而当抗PD-1/PD-L1联合抗血管生成治疗对比抗PD-1/PD-L1联合化疗从患者获益角度来看似乎无明显的统计学差异，但真实世界结果如何尚未知。

## 4 优化获益人群

目前仅从PD-L1表达情况、TMB来预测抗PD-1/PD-L1疗效已不能满足临床需求。

IMpower150研究^[50]^表明ABCP组较BCP组均有不同程度OS的改善，这与患者PD-L1的表达状态无关。Shen等^[44]^及Yu等^[45]^发现了T790M突变对*EGFR*突变的晚期NSCLC患者接受EGFR-TKI耐药后使用免疫联合化疗的疗效的负面影响。Ye等^[53]^研究发现28例*EGFR*突变的非鳞NSCLC患者中，7例患者肿瘤比例评分（tumor proportion score, TPS）≥50%。Yamada等^[54]^通过对27例*EGFR*突变NSCLC患者免疫检查点抑制剂（immune checkpoint inhibitors, ICIs）的回顾性疗效分析发现*EGFR*罕见突变（包括外显子18中的G719X和外显子20插入）患者的ORR和疾病控制率（disease control rate, DCR）高于*EGFR*常见突变患者（分别为71% *vs* 35.7%、57% *vs* 7%，*P*=0.14、*P*＜0.01），罕见*EGFR*突变或无T790M突变的患者的中位PFS明显更长（分别为*P*=0.003、*P*=0.03）。Ozaki等^[55]^研究发现，基于多变量分析*EGFR*突变阴性（*P*=0.011,1）和*TP53*突变阳性（*P*=0.042,5）TMB升高。Chen等^[56]^研究发现*EGFR* Ex20ins患者的PD-L1表达显著高于*HER2*突变患者（48.6% *vs* 19.0%, *P*=0.027），高TMB（*P*=0.025）和PD-L1（*P*=0.045）表达与预后不良独立相关，而CD4 /CD8^+^ TILs与*EGFR*或*HER2*突变型NSCLC的预后之间没有关联，*EGFR* Ex20ins患者的PD-L1表达显著高于*HER2*突变患者，而这可能是对抗PD-1/PD-L1治疗应答不同的潜在原因之一。Shi等^[57]^研究发现，对比野生基因型，*EGFR*致敏突变、*ALK*重排、*ROS1*重排TMB较低，在PD-L1^+^/TMB-H组所占比例低。Metro等^[58]^对30名*EGFR* Ex20ins突变的晚期NSCLC患者进行了研究，其中15患者接受了免疫治疗，发现接受ICIs治疗的患者与未接受免疫治疗的患者相比，有较短的中位OS（分别为12.9个月、25.2个月，*P*=0.08），在多变量分析中，这种差异与较差的生存结果相关（*P*=0.04）。

从以上研究可以看出不同基因改变影响肿瘤的免疫状态，同一基因的不同亚型改变对抗PD-1/PD-L1应答也不同。EGFR-TKI耐药后会出现继发基因改变，不同的基因改变对免疫应答如何了解甚少。目前认为*TP53*、*KRAS*突变对抗PD-1/PD-L1有相对较好的应答，当*EGFR*突变合并*TP53*或*KRAS*突变抗PD-1/PD-L1疗效有何变化缺乏大规模研究。有研究^[59]^表明*EGFR*突变等位基因频率可预测EGFR-TKI的疗效，类似地，*EGFR*突变等位基因频率是否对抗PD-1/PD-L1有预测作用仍未知。

## 5 总结与展望

抗PD-1/PD-L1治疗很大程度上改变了肿瘤治疗的格局，但大部分*EGFR*突变的NSCLC患者对抗PD-1/PD-L1治疗低应答或无应答，这可能是因为*EGFR*突变使NSCLC的TME为免疫排除型或免疫沙漠型，研究其内在机制对TME转为免疫炎症型有重要意义。*EGFR*突变的NSCLC患者的抗PD-1/PD-L1联合化疗和/或抗血管治疗有协同作用，但并非所有患者产生临床获益，仍需寻找可靠的标志物来预测疗效。总之，在精准医疗的时代，基因改变对免疫功能及相关药物疗效的影响研究仍有很大空间。
